# Association between Urinary Albumin Excretion and Intraocular Pressure in Type 2 Diabetic Patients without Renal Impairment

**DOI:** 10.1371/journal.pone.0096335

**Published:** 2014-05-02

**Authors:** Jin A. Choi, Kyungdo Han, Hyuk-Sang Kwon

**Affiliations:** 1 St. Vincent Hospital, Department of Ophthalmology, Division of Endocrinology and Metabolism, Department of Internal Medicine, College of Medicine, The Catholic University of Korea, Seoul, Republic of Korea; 2 St. Vincent Hospital, Department of Biostatistics, Division of Endocrinology and Metabolism, Department of Internal Medicine, College of Medicine, The Catholic University of Korea, Seoul, Republic of Korea; 3 Yeouido St. Mary's Hospital, Division of Endocrinology and Metabolism, Department of Internal Medicine, College of Medicine, The Catholic University of Korea, Seoul, Republic of Korea; IRCSS - Istituto di Ricerche Farmacologiche Mario Negri, Bergamo, Italy

## Abstract

**Background:**

To assess the relationship between urinary albumin excretion and intraocular pressure (IOP) in type 2 diabetes patients without renal impairment.

**Methods:**

We explored the effects of albuminuria on high IOP in 402 non-glaucomatous type 2 diabetes without renal impairment who participated in the 2011 Korean National Health and Nutrition Examination Survey (KNHANES). Multiple logistic regression analysis was used to assess the relationship between log-transformed albumin/creatinine ratio (ACR) tertiles and an IOP of ≥18 mmHg after adjusting for age, gender, hypertension, body mass index, triglycerides, area of residence, and education level.

**Results:**

Subjects with a high IOP ≥18 mmHg were more likely to be current smokers (P = 0.038), heavy drinkers (P = 0.006), and to have high systolic blood pressure (P = 0.016), triglycerides (P = 0.008), and a higher log-transformed ACR (P = 0.022).In multivariate regression analysis, ACR tertile was associated with the prevalence of high IOP significantly (*P* = 0.022). The associations between ACR tertiles and high IOP were significant in overweight patients and those with abdominal obesity (P = 0.003 and 0.003, respectively). In contrast, there were no associations in the subgroup of patients who were not overweight and those without abdominal obesity (P = 0.291 and 0.561, respectively).

**Conclusions:**

Urinary albumin excretion is associated with high IOP in the type 2 diabetes population without renal insufficiency. The effect of the albuminuria on IOP was evident in a subgroup of patients with components of metabolic syndrome.

## Introduction

Glaucoma is one of the most common causes of blindness, and intraocular pressure (IOP) is the only known modifiable risk factor for glaucoma [1]. Recent reports showed that elevated IOP was associated with metabolic syndrome, which includes clustered metabolic disorders such as abdominal obesity, dyslipidemia, hypertension, and hyperglycemia [2], [3]. Insulin resistance, the common underlying mechanism for type 2 diabetes and metabolic syndrome, may be a key factor that contributes to elevated IOP in these conditions. Type 2 diabetes is also associated with elevated IOP, and many reports have suggested that diabetes may be one of the risk factors for glaucoma [4]–[6].

Microalbuminuria is an important prognostic marker for kidney disease during diabetes or hypertension [7]–[9]. It is also a marker for vascular endothelial dysfunction, and so could be used as an indicator of subclinical cardiovascular diseases [10], [11]. Therefore, the World Health Organization (WHO) includes microalbuminuria as a diagnostic criterion for the metabolic syndrome [12]. However, the association between albuminuria and IOP in type 2 diabetes mellitus has not yet been investigated. The aim of this study was to investigate the relationship between urinary albumin excretion and IOP in patients with type 2 diabetes without renal impairment.

## Patients and Methods

### Data source and participants

The Korea National Health and Nutrition Examination Survey (KNHANES) is a nationwide, population-based, cross-sectional health examination and survey conducted regularly by the Division of Chronic Disease Surveillance, Korea Centers for Disease Control and Prevention, Ministry of Health and Welfare, to monitor the general health and nutritional status of individuals in South Korea. KNHANES was performed in 1998 (KNHANES I), 2001(KNHANES II), 2005 (KNHANES III), 2007–2009 (KNHANES IV), and 2010–2012 (KNHANES V). It consists of a health interview survey, a nutritional survey, and a health examination survey. A stratified, multistage probability sampling design is used to select the households that participate in the survey. KNHANES was performed according to the guidelines of the Declaration of Helsinki. All participants in the survey signed an informed consent form. The Institutional Review Board of St. Vincent Hospital, College of Medicine, the Catholic University of Korea, Seoul, Korea approved the protocol.

The present study is based on data from KNHANES 2011 to estimate the association between albumin excretion and IOP in type 2 diabetic patients without renal impairment. In KNHANES 2011, a total of 8,055 out of 10,589 (76.1%) individuals participated in the survey. 727 subjects with missing estimated glomerular filtration rate (eGFR) or urine albumin-to-creatinine ratio (ACR) data were excluded. A total of 5,349 subjects aged ≥20 years were assessed using laboratory testing and nutritional survey data, and those with diabetes (fasting glucose concentration ≥126 mg/dL, or prescribed insulin or oral anti-diabetic medication; n = 567) were included. We then excluded subjects with baseline glaucoma (n = 19), intraocular surgery including vitrectomy or cataract surgery (n = 93), and renal insufficiency with an eGFR <60 mL/min/1.73m2 (n = 53) [7]. After these exclusions, the final study population included 402 subjects aged ≥20 years (226 males and 176 females).

### Measurements

Blood samples were collected from each participant in the morning following an overnight fast. Samples were processed, refrigerated immediately, and transported in cold storage to the Central Testing Institute in Seoul, Korea. All blood samples were analyzed within 24 h of transportation. The concentration of glucose, total cholesterol, and triglycerides were assessed using a Hitachi automatic analyzer 7600 (Hitachi, Tokyo, Japan). The levels of glycated hemoglobin (HbA1C) were measured using high-performance liquid chromatography. The estimated glomerular filtration rate (eGFR) was calculated using the modification of diet in renal disease study equation [13]. The albumin/creatinine ratio (ACR) was calculated for first-voided spot urine samples. Subjects were categorized into ACR tertiles with cut-off values that were determined separately for males and females.

Demographic variables, including age, gender, area of residence, income status, and education level, were recorded. The region of habitation was categorized as urban or rural. Among the 16 districts of South Korea, eight major cities (Seoul, Gyeonggi, Busan, Daegu, Incheon, Gwangju, Daejeoun, and Ulsan) were grouped as urban areas, and the remaining provinces (Gangwon, Chungbuk, Chungnam, Jeonbuk, Jeonnam, Gyeongbuk, Gyeongnam, and Jeju) were grouped as rural areas. Participants were categorized in the low-income group if their income was in the lowest quartile. Data pertaining to current smoking (a person who smokes cigarettes daily) were also recorded. For alcohol consumption, a heavy drinker was defined as an individual who consumed four or more drinks per week. Information regarding the duration of diabetes and any prescribed medications was also obtained. Physical activity was measured by self-reporting using the International Physical Activity Questionnaire [14]. Moderate physical activity was categorized as “yes” when participants engaged in moderate-intensity physical activity for >20 min three or more times per week. Moderate-intensity physical activity was defined as causing a slight increase in breathing or heart rate for at least 10 min, such as when carrying light loads, cycling at a regular pace, or playing tennis.

A specially trained examiner recorded anthropometric measurements. Blood pressure (BP) was measured in the right arm using a standard mercury sphygmomanometer (Baumanometer, WA Baum Co., New York, USA) after 5 min of rest in the sitting position. Systolic and diastolic BPs were measured three times at 30-s intervals; the second and third measurements were then averaged to produce the final BP used for analysis. Hypertension was defined as a systolic hypertension ≥140 mmHg or diastolic pressure ≥ 90 mmHg, or the use of antihypertensive medication. Height was measured to the nearest 0.1 cm using a portable stadiometer (SECA 225, SECA Deutschland, Hamburg, Germany) when participants were standing barefoot. Bodyweight was measured to the nearest 0.1 kg on a balanced scale (GL-6000-20, CAS KOREA, Seoul, Korea) while participants wore a lightweight gown. BMI was calculated as the individual's weight in kilograms divided by the square of their height in meters. Subjects were categorized as lean (BMI <25 kg/m2) or overweight (BMI ≥ 25 kg/m2). Waist circumference was measured using a measuring tape (SECA 200, SECA Deutschland) to the nearest 0.1 cm in a horizontal plane at the midpoint between the iliac crest and the costal margin at the end of normal expiration. The presence of abdominal obesity, a measure of metabolic syndrome, was recorded based on a waist circumference >90 cm in males or >80 cm in females.

Ophthalmologic examinations were performed by ophthalmologists using a slit lamp (Haag-Streit model BQ-900; Haag-Streit AG, Koeniz, Switzerland). The IOP in both eyes was measured using a Goldmann applanation tonometer. Because the IOP in the right and left eyes was highly correlated (Pearson'scorrelation  = 0.86, P<0.001), only data for the right eyes were used.IOP was categorized as low (IOP <18 mmHg) or high (IOP ≥18 mmHg) [15]. In each participant, macula-and disc-centered views (seven standard photographs from the Early Treatment for Diabetic Retinopathy Study) [16] were taken at an angle of 45° using a fundus camera after pharmacological mydriasis. The fundus photographs were evaluated by trained graders who were unaware of the patient characteristics, based on photographic standards [6], [17]. The presence of any grade of diabetic retinopathy was recorded for each participant.

### Statistical analyses

Statistical analyses were performed using the SAS survey procedure (version 9.2; SAS Institute, Inc., Cary, NC, USA) to reflect the complex sampling design and sampling weights of KNHANES, and to provide nationally representative prevalence estimates. Procedures included unequal probabilities of selection, oversampling, and non-response so that inferences could be made for all Korean participants.

Participant characteristics were described using means and standard errors for continuous variables, and numbers and percentages for categorical variables according to standard error. Logarithmic transformation was used to analyze variables with skewed distribution, such as TG and ACR. General and clinical characteristics were compared between subjects with high and low IOP. The prevalence of subjects with high IOP in different ACR tertiles was also determined. Multiple logistic regression analysis (including age, gender, the presence of hypertension, BMI, TG, blood glucose level, region of habitation, and education level) was used to examine the association between the ACR tertile and the prevalence of high IOP. Next, patient subgroups were analyzed based on those that were overweight (BMI ≥25 kg/m2 or <25 kg/m2) or with abdominal obesity (based on waist circumference). In each subgroup, the prevalence of high IOP was determined across the ACT tertiles. Finally, the odds ratios (ORs) for each ACR tertile and the presence of high IOP were calculated in each subgroup.

## Results

**Figure 1:**
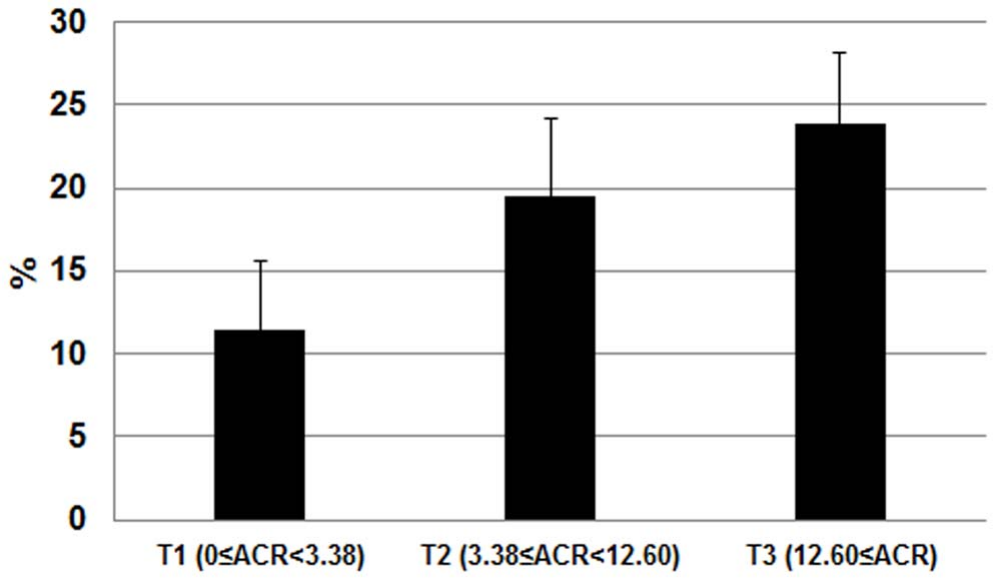
Prevalence of subjects (%) with high intraocular pressure (IOP; ≥18 mmHg) separated by albumin/creatinine ratio (ACR) tertiles (P for trend  = 0.020).

The study group consisted of 402 non-glaucomatous participants with type 2 DM, but without renal impairment. The mean age of the subjects was 55.43±0.86 years, and the mean BMI was 24.96±0.23 kg/m2. The mean hemoglobin A1c was 7.37±0.11%, and the mean duration of DM was 5.04±0.37 years. A comparison of the clinical and biochemical parameters of subjects with high (≥18 mmHg) and low IOP (<18 mmHg) is shown in Table 1. Subjects with high IOP were more likely to be current smokers (P = 0.038), current heavy drinkers (P = 0.006), have high systolic BP (P = 0.016), elevated TG (P = 0.008), and marginally high fasting serum glucose (P = 0.098). There was a significant difference in the log-transformed ACR (P = 0.022). The tertile cutoff values of ACR were as follows: T1 <3.38, 3.38≤T2<12.60, and T3 ≥12.60. The prevalence of subjects with high IOP was increased significantly in higher ACR tertiles (P = 0.020) (Figure 1). The results of multiple logistic regression analyses are shown in Table 2. ACR tertile was associated with the prevalence of high IOP significantly after adjusting for age and gender (P = 0.024); this was maintained after adjusting for the presence of hypertension, BMI, triglycerides, area of residence, and education level (P = 0.022). Additional adjustment for blood glucose level reduced the magnitude of the odds ratio for ocular hypertension, but did not affect their statistical significance (P = 0.043).

**Table 1:** Clinical characteristics of study subjects according to the intraocular pressure in Korean type 2 diabetic patients without renal impairment (n = 402).

	Subjects with Low IOP	Subjects with High IOP	
	IOP<18 mmHg	IOP≥18 mmHg	*P*-value
Number	334	68	
Age (year)	55.66±0.95	54.38±1.74	0.507
Gender (% of female)	38.1	35.4	0.707
IOP (mmHg)	13.74±0.17	18.72±0.13	**<0.001**
Height (cm)	163.12±0.51	164.42±1.63	0.443
Weight (kg)	67.05±0.76	66.46±1.84	0.769
Waist circumference (cm)	87.58±0.71	86.39±1.42	0.462
BMI (kg/m^2^)	25.07±0.25	24.52±0.56	0.372
Fasting Serum Glucose (mg/dL)	142.56±2.90	157.80±8.75	**0.098**
HbA1C ( %)	7.31±0.11	7.65±0.26	0.246
DM duration (≥ 5yr)	39.6	33.4	0.564
Diabetic retinopathy (yes, %)	15.4	10.4	0.298
History of hypertension (yes, %)	22.6	21.1	0.832
Medical Treatment for DM (yes, %)	62.6	58.2	0.371
Systolic BP, mmHg	123.89±1.27	132.40±3.11	**0.016**
Diastolic BP, mmHg	77.37±0.58	80.47±1.53	**0.066**
Serum total cholesterol	186.35±2.77	195.52±5.51	0.144
Serum triglyceride (mg/dL) a	136.81 (124.21–150.67)	182.97 (152.16–229.99)	**0.008**
Serum creatinine (mg/dL)	0.86±0.01	0.85±0.03	0.789
GFR (mL/min/1.73 m^2^)	92.08±1.32	93.76±2.37	0.541
Urine albumin (µg/mL)	35.63±6.38	374.67±235.45	0.152
ACR^a^	8.72 (7.51–10.13)	18.8 (9.60–36.66)	**0.022**
Smoker (current, %)	28.4	46.0	**0.038**
Current drinker (heavy, %)	12.0	29.7	**0.006**
Education level (≥ 9y, %)	68.6	69.6	0.877
Area of residence (Rural, %)	27.5	28.2	0.914
Low income (lowest quartile, %)	22.7	21.1	0.819
Regular physical activity (≥ mod intensity, %)	16.8	19.3	0.673

Data are presented as mean ± SE or as % (SE).

a Geometric mean (95% CI).

**Table 2:** Association between ACR and prevalence of high IOP (18 mmHg or more) in Korean type 2 diabetic population without renal impairment (n = 402).

	Model 1:	Model 2:	Model 3:
	unadjusted	adjusted for age and gender	adjusted for age, gender, hypertension, BMI, TG, habitation, and education
ACR			
T1(ACR≤3.38)	1	1	1
T2(3.38<ACR≤12.60)	1.88(0.68–5.25)	1.95(0.66–5.76)	1.93(0.69–5.37)
T3(12.60<ACR)	2.43(1.02–5.77)	2.64(1.06–6.55)	2.6(1.09–6.19)
P for trend	0.032	0.024	0.022

**Figure 2:**
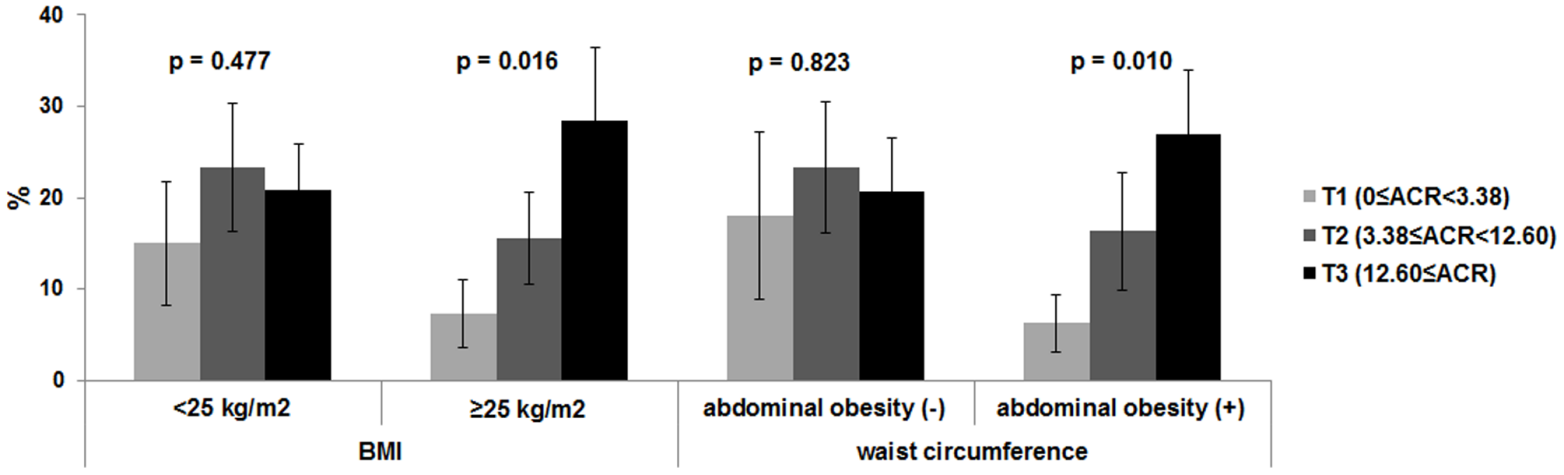
The relationships between albumin/creatinine ratio (ACR) tertiles and the prevalence of high intraocular pressure (IOP; ≥18 mmHg) in subgroups based on body mass index and abdominal obesity. The P values for trends are shown.

**Figure 3:**
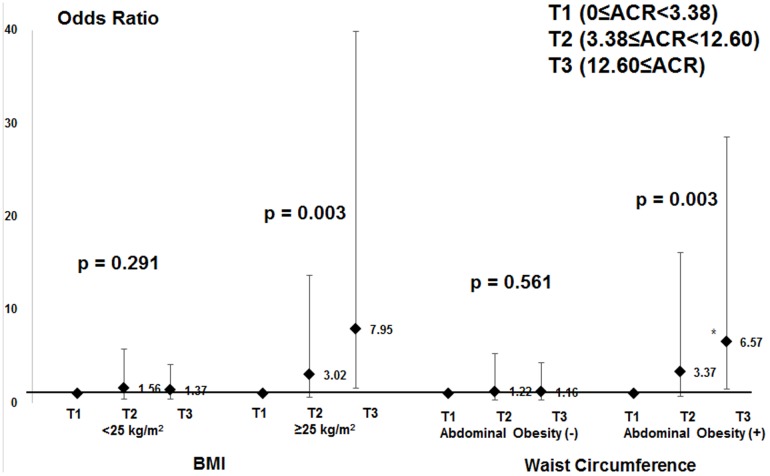
Adjusted odds ratio for high intraocular pressure (IOP; ≥18 mmHg) according to albumin/creatinine ratio (ACR) tertiles in subgroups based on body mass index and waist circumference. The error bars represent 95% confidence intervals. Odds ratios were estimated using a multiple logistic regression analysis after adjusting for age, gender, hypertension BMI, triglycerides, region of habitation, and education level. The P values for trends are shown.

Figure 2 shows the prevalence of high IOP across the ACR tertiles in patient subgroups that were overweight or with abdominal obesity. In the overweight subgroup, the prevalence of high IOP was increased significantly according to ACR tertile (P for trend = 0.016), whereas no trend was observed in non-overweight patients (P = 0.477). Similarly, in the patient subgroup with abdominal obesity the prevalence of high IOP was increased significantly according to ACR tertile (P = 0.010). In contrast, there was no trend in the patient subgroup without abdominal obesity (P = 0.823). Figure 3 shows that subjects in the highest ACR tertile, particularly those that were overweight or with abdominal obesity, were significantly more likely to have high IOP compared with those in the two lower ACR tertiles. In contrast, subjects in the highest ACR tertiles that were not overweight and without abdominal obesity exhibited no statistically significant associations with high IOP.

## Discussion

In the present study, urinary albumin excretion was associated with a high IOP of ≥18 mmHg, independent of age, gender, hypertension, BMI, dyslipidemia, blood glucose level, region of habitation, and education level. In addition, the association between ACR and IOP was distinct in subgroups of patients with components of metabolic syndrome. Collectively, these findings suggest that albuminuria might be a risk factor for increased IOP in diabetic patients without renal impairment.

It is well known that diabetes is associated with elevated IOP [4]–[6]. Although several risk factors such as HbA1C have been suggested, the causative factors associated with high IOP in diabetic patients have not yet been elucidated. Albuminuria is a well-known marker of nephropathy in patients with type 2 diabetes [5], [8], [18]. Recent studies have emphasized the role of albuminuria as an indicator of cardiovascular outcome and a marker of vascular endothelial dysfunction [19]–[23]. To our knowledge, this is the first study to assess the role of albuminuria as a predictor of high IOP in diabetic patients.

The exact mechanism by which albuminuria might be associated with higher IOP is unknown. One reason for the positive correlation between ACR and IOP might be that albuminuria is associated with the presence of metabolic syndrome [23], [24]. In addition, metabolic syndrome and other insulin resistance-related characteristics were strongly associated with high IOP [3]. Consistent with this, our study identified that the correlation between ACR and IOP was prominent in subpopulations that were overweight or with abdominal obesity (Figs. 2 and 3). These findings suggest that ACR might play an independent role in ocular hypertension, as well as metabolic syndrome.

The presence of albuminuria was associated with biochemical indexes of endothelial dysfunction, such as increases in the serum levels of von Willebrand factor, endothelin, tissue plasminogen activator, and fibrinogen [25]. From a structural point of view, the anterior chamber could be regarded as a specialized circulatory vessel lined with endothelial cells of the corneal and trabecular endothelium. The endothelial cells that line the channels of the trabecular meshwork and Schlemm's canal are part of the pathway for aqueous humor outflow from the eye; the trabecular meshwork shows smooth-muscle-like contractility [26]. Endothelin was implicated in the pathogenesis of glaucoma, and levels were increased in the aqueous humor of glaucoma patients [27], [28]. This causes contraction of the trabecular meshwork cells, decreasing the inter-trabecular space and thereby increasing outflow resistance. The decreased aqueous outflow caused by endothelial dysfunction and disruption of aqueous humor homeostasis may result in fluid overload, increasing IOP. To confirm this hypothesis, further studies are required, because this is cross-sectional study and we did not directly address this issue in this study. Additionally, we included only non-glaucomatous eyes to exclude any potential confounding effects on IOP. Therefore, the association between ACR and glaucoma should be considered in a future study.

Consistent with our findings, the Singapore Malay Eye study reported that the presence of chronic kidney disease was associated with higher IOP, independent of age, diabetes, and glaucoma [29]. Diabetic retinopathy and nephropathy, the major microvascular complications of diabetes, are closely interrelated, and progress in a parallel manner [17]. As diabetic retinopathy progresses, secondary glaucoma can be complicated. A previous study demonstrated a consistent association between neovascular glaucoma and proliferative retinopathy, which was the most common cause of this secondary glaucoma [30]. However, the present study revealed that the association of albuminuria with IOP was significant in diabetes patients without renal insufficiency (eGFR ≥60 mL/min/1.73 m2). The fact that albuminuria was independently associated with increased IOP in patients without kidney disease suggests that urinary albumin excretion might be an independent and more relevant pathophysiological surrogate of increased IOP than GFR.

Classically, the term ‘ocular hypertension’ is used to distinguish individuals with normal IOP from those with an IOP >21 mmHg. However, in a recent Korean epidemiological study, 77% of the patients with primary open-angle glaucoma had an IOP <21 mmHg [31]. This was consistent with the data reported by Tajimi [32] and Handan [33], in which ∼90% of primary open-angle glaucoma patients had an IOP <21 mmHg. This suggests that the criteria for ocular hypertension might be lower than 21 mmHg, particularly in East Asian populations. In the advanced glaucoma intervention study, eyes with IOP <18 mmHg in 100% of visits had almost no deterioration in the visual field defect score over 6 years [15], suggesting that an IOP <18 mmHg is tolerable in most glaucoma patients. In accordance with this, participants were categorized as having high IOP based on a baseline IOP of ≥18 mmHg in this study.

The present study is the first to identify the association between albuminuria and increased IOP in type 2 diabetic populations without renal insufficiency. The data have great significance because they used nationally representative data. However, some limitations must be acknowledged. Because of the cross-sectional and retrospective study design, the association between ACR and IOP may not imply a causal relationship. ACR is known to have high intra-individual variation, particularly in the intermittently microalbuminuric patients [34]. High variability in ACR might have biased the results, considering that a single measurement is considered in this study. The study may also be confounded by a lack of data on additional relevant variables such as several components of metabolic syndrome, including HDL cholesterol and LDL cholesterol. The Goldmann applanation tonometry used in this study is the gold standard for measuring IOP. However, this is affected by central corneal thickness, albeit to a lesser extent than noncontact pneumotonometry [35]. The association between ACR and IOP would have been more accurate if the central corneal thickness was measured and included in the analyses.

In summary, this study demonstrated that urinary albumin excretion is associated with high IOP in type 2 diabetes patients without renal insufficiency. The effect of the albuminuria on IOP was evident in patient subgroups with components of metabolic syndrome. These data highlighted a potential role of albuminuria in ocular hypertension in diabetic patients.
